# The role of salicylic acid and benzothiadiazole in decreasing phytoplasma titer of sugarcane white leaf disease

**DOI:** 10.1038/s41598-021-94746-9

**Published:** 2021-07-26

**Authors:** Manuwat Tintarasara Na Ratchaseema, Lawan Kladsuwan, Laurent Soulard, Pratchya Swangmaneecharern, Prapat Punpee, Peeraya Klomsa-ard, Klanarong Sriroth, Suttipun Keawsompong

**Affiliations:** 1 Mitr Phol Sugarcane and Research Center, 399 M1, Koksa-ad, Phukhieo, 36110 Chaiyaphum Thailand; 2 Department of Biotechnology, Faculty of Agro-Industry, Kasetsart University, Bangkok, 10900 Thailand

**Keywords:** Biotechnology, Molecular biology, Plant sciences

## Abstract

The objective of this research was to study the effect of Benzothiadiazole (BTH) and Salicylic acid (SA) on the systemic acquired resistance (SAR) of sugarcane the phytoplasma associated with the sugarcane white leaf (SCWL) disease. The experiment was conducted on plants of the sugarcane variety Khon Kaen 3 (KK3) infected with SCWL phytoplasma using insect vectors. Biochemical changes related to the SAR such as SA and total phenolic compounds were followed according to 4 different timepoints: 7, 14, 21 and 28 days after inoculation. Together, phytoplasma were quantified by RT-qPCR using the *secA* gene of phytoplasma. According to our results, the spraying of BTH and SA tended to increase the amounts of SA, total phenolic compounds and a lower presence of phytoplasma in the plants in comparison with the inoculated control. Spraying BTH at a concentration of 2.4 mM and SA at a concentration of 2.4 mM exhibited the best efficiency to reduce the concentration of phytoplasma. According to RT-qPCR results, the inoculated plants sprayed with BTH displayed a significantly lower concentration of phytoplasma compared to the inoculated controls. Overall, our results indicated that the spray of BTH and SA could induce an efficient SAR response to the phytoplasma associated with the SCWL disease. We expect these results will give support to the development of new products for controlling white leaf disease in sugarcane.

## Introduction

Sugarcane (*Saccharum officinarum* L.) is one of the most important economic crops in Thailand with an area of 1.91 million hectares throughout the country and a production of 74.89 million tons of sugarcane during the year 2019/20^1^. Sugarcane is widely used for various applications in food and feed industries and for industrial purposes. In 2011/12, more than 27,200 hectares of sugarcane plantation were found to be infected by the phytoplasma associated with SCWL (16SrXI group), mainly in the northeastern region of Thailand, and resulting in an economic loss for sugarcane farmers and the sugar industries estimated at approximately one billion baht^2^. Several factors were associated with the outbreak of the disease within Thailand sugarcane plantation; a low-abundance of nutrient in the soil, a sandy soil type suitable for the reproduction of insect vectors^3,4^ and this problem was found in the northeastern region of Thailand in 2007/08 and 2011/12 more than 27,000–29,000 hectares^2^, and a large majority of fields covered with SCWL-susceptible varieties such as KK3. The phytoplasma is spread by insect vectors such as the leafhopper species *Matsumuratettix hiroglyphicus* and *Yamatotettix flavovittatus*^4,5^. SCWL symptoms manifest as pale leaves due to loss of chlorophyll; the leaves are small, short, broken, and shredded^6^.

Currently, several methods are used to control the SCWL such as the hot-water treatment of cuttings before planting^7^, disease-free sugarcane from tissues culture^8^, the removal of infected plants from the field, crop rotations and the application of biopesticide to control the population of insect vectors^9^. Recent research works have been focusing on the use of chemical elicitors to boost plant resistance and strength against diseases and insect vectors such as using biotic elicitors by implanting pathogens that are less severe for the plants^10^, or abiotic elicitors where chemical compounds^11^, organic or inorganic substances, are from certain microorganisms^12^. Many studies revealed that the application of both abiotic, i.e., salicylic acid (SA), ascorbic acid, benzo (1, 2, 3) thiadiazole-7-carbonic acid S-methyl ester (BTH), DL-β-aminobutyric acid (BABA) and acibenzolar-S-methyl (ASM), and biotic elicitors, i.e., *Bacillus subtilis*, *Pseudomonas sp*. and *Trichoderma sp.*^13^ at appropriate level induced a disease resistance in several plants able to limit or inhibit the development of fungal^14,15^, viral^16^ and bacterial pathogens^17^. During the infection of the plant by a pathogen, phytohormones such as Salicylic acid, jasmonic acid and ethylene are widely produced by the plants in response to the infection to limit the development of the pathogen^18,19^. Chemical elicitors are able to stimulate the plant immunity and the accumulation of phytoalexins and proteins associated with plant resistance such as pathogenesis-related (PR) proteins (e.g., chitinase and β-1, 3-glucanase enzymes)^12^. This inhibition system is called induced systemic resistance (ISR)^20^ or systemic acquired resistance (SAR)^21^, where the SAR system uses SA as a signaling molecule to trigger the accumulation of PR proteins^22^.

The effect of the elicitors SA and BTH on the resistance to a wide range of pathogens including phytoplasma, have been intensively studied^23–26^. The spraying of 100 mM BTH on Arabidopsis could stimulate the SAR with high levels of the PR-1 gene expression^23^. The BTH reduced crown rot, caused by Phytophthora cactorum in strawberries^27^. The treatment after spraying BTH for 7 days and before implanting the CMV-Y virus was demonstrated that decreased in the incidence of tomatoes^28^. Besides, the BTH was used for stimulating a resistance to a red rot disease in sugarcane^29^. BTH minimized the diffusion of bacteria within the cane tissues, the resistance induced (SAR) lasted for about 30 days after the stimulation. It was reported that spraying BTH at a concentration of 250 µg/mL induced the production of phenolic compounds and the accumulation of β-1, 3-glucanase and thaumatin-like protein (PR-5). The glycoprotein from *Colletotrichum falcatum* induced a SAR systemic resistance by increasing the activity of chitinase and β-1,3-glucanase enzymes in sugarcane varieties resistant to red rot disease (BO 91) compared to susceptible varieties (CoC 671)^30^. The BTH and SA showed the resistance against red rot disease in field conditions using susceptible sugarcane varieties. Moreover, the expression of the disease symptoms was reduced by using the BTH and SA application^31^. The BTH treatment induce through the SA dependent partway effect to delay in phytoplasma multiplication and disease development^32^. In addition, the BTH and two glutathione-plus-oligosaccharide compounds could significantly induce a recovery from the Bois noir disease in grapes^26^. The spraying of BTH solution at 4.8 mM reduced the infection from insect vectors in *Arabidopsis thaliana* by approximately 50%^24^. In addition, the study of BTH was reported the prevention of the Flavescene dorée (FDP) phytoplasma transmission infection in grapevines^33^. Whilst the BTH application at 2.4 mM could reduce the severity of disease from phytoplasma in daisy trees^25^. The application of SA at a concentration of 0.1 mM and 0.001 mM in potato that can against phytoplasma, decrease infection, high photosynthate translocation and improved high yield^34,35^. Moreover, the tomato can be against 53% of potato purple top phytoplasma infection after treated with SA^36^. Although many studies have been published on the usage of elicitors against phytoplasma diseases in several plants, the effect in sugarcane against the SCWL disease has not been reported.

In this research, we investigated the effect of SA and BTH at various concentration on the SCWL disease in sugarcane, inoculated by *M. hiroglyphicus* (Matsumura) insect vector. The presence of phytoplasma in plants after inoculation was quantified. Furthermore, the effect of stimulants on the synthesis of biochemical substances related to SAR in the plant and their impact on the expression of SCWL symptoms were investigated. This work aims to define suitable concentrations of stimulants for a large-scale control of the SCWL.

## Results

This experiment studied the effect of spraying SA and BTH against the SCWL disease in sugarcane. Sugarcane leaves were sampled from control groups and sugarcanes treated with SA or BTH and then inoculated with SCWL phytoplasma. The amount of phytoplasma in our samples was quantified by RT-qPCR using the *secA* gene.

**Figure 1:**
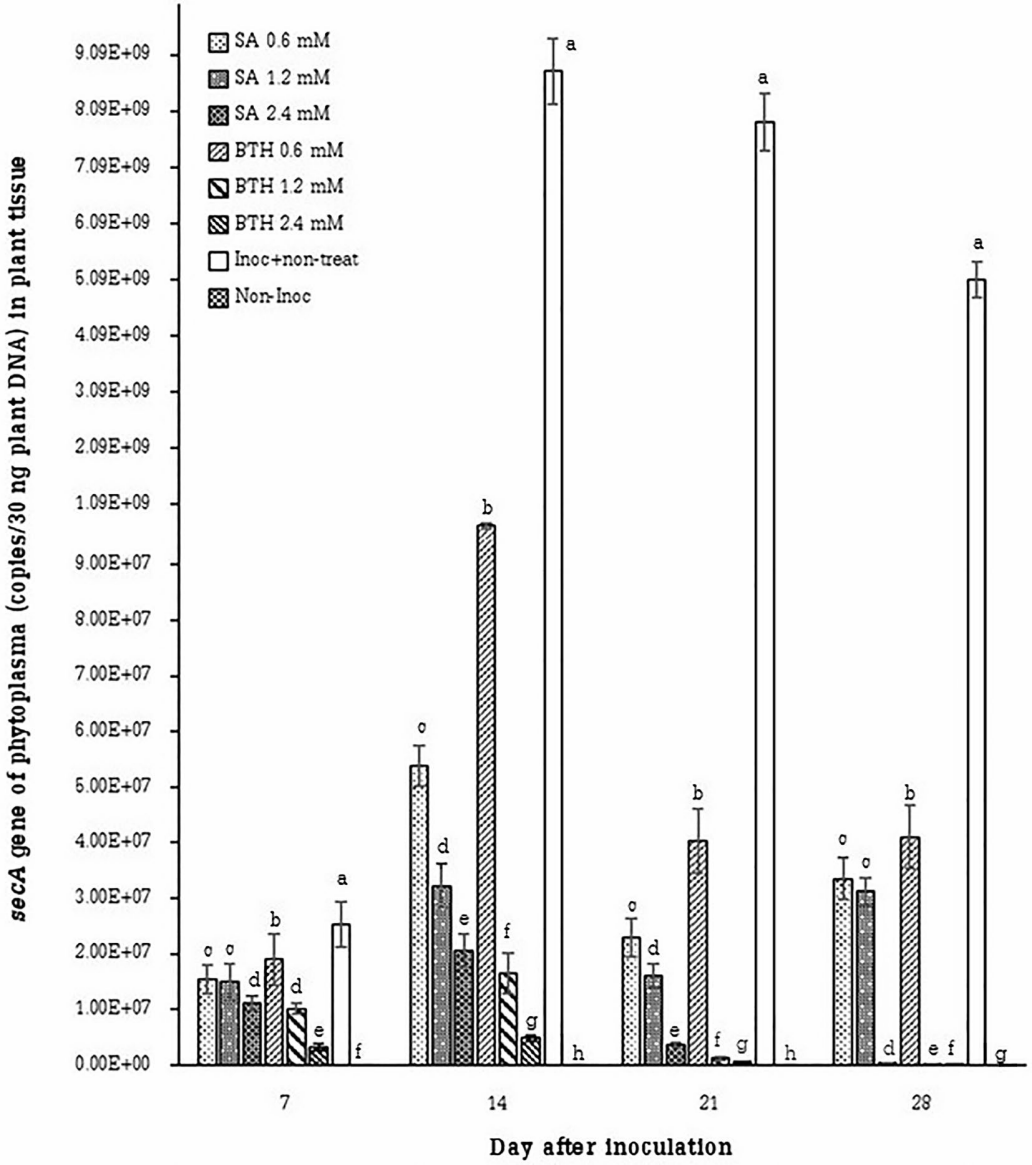
Quantification of the gene *secA* (copies/30 ng plant DNA) in sugarcane cv. KK3 by RT-qPCR technique. Data was collected at timepoints 7, 14, 21, and 28 days after inoculation (n=10 in each treatment).

According to our results, no significant differences were observed in the presence of phytoplasma before and after 4 weeks of SA and BTH treatments (Table 1). In addition, the concentration of phytoplasma in plants treated with SA and BTH was significantly lower than the inoculated nontreated control plants at each timepoint (Fig. 1). According to RT-qPCR results, the spray of BTH at a concentration of 2.4 mM was the treatment showing the best performance the phytoplasma associated with the SCWL disease, since it was showed lower concentrations of phytoplasma. The concentrations of phytoplasma were estimated at 3.20 × 10^6^, 4.82 × 10^6^, 5.72 × 10^5^ and 3.47 × 10^3^ copies/30 ng plant DNA after transplantation for timepoints 7, 14, 21 and 28 days, respectively. Moreover, for a treatment with BTH at a concentration of 1.2 mM, concentrations of phytoplasma were estimated at 1.01 × 10^7^, 1.65 × 10^7^, 1.16 × 10^6^ and 1.60 × 10^5^ copies/30 ng plant DNA at timepoints 7, 14, 21 and 28 days, respectively. Furthermore, for the treatment with SA at a concentration of 2.4 mM, concentrations of phytoplasma were measured at 1.12 × 10^7^, 2.06 × 10^7^, 3.63 × 10^6^ and 3.59 × 10^5^ copies/30 ng plant DNA for timepoints 7, 14, 21 and 28 days, respectively. The inoculated control showed the highest concentrations of phytoplasma with 2.53 × 10^7^, 8.80 × 10^9^, 7.89 × 10^9^ and 5.09 × 10^9^ copies/30 ng plant DNA after 7, 14, 21 and 28 days, respectively.

**Table 1:** Quantification of the gene *secA*, salicylic acid and total phenolic compound in control groups (inoculated control and non-inoculated control), before (the sampling time 1) and after spraying the elicitors (the sampling time 2) in sugarcane treated groups.

Treatment	*secA* gene of phytoplasma (copies/30 ng plant DNA)		Salicylic acid (SA) (µg g^−1^ sample)		Total phenolic compound (µg GAE mg^−1^ sample)	
	*Before treated	*After treated	*Before treated	*After treated	*Before treated	*After treated
SA 0.6 mM	4.31 ± 0.93	4.25 ± 0.52	42.85 ± 2.65	47.31 ± 2.64 ^bc^	46.47 ± 3.14	49.88 ± 1.38 ^ab^
SA 1.2 mM	4.27 ± 0.74	4.13 ± 0.88	41.90 ± 0.38	47.38 ± 0.67 ^bc^	47.17 ± 1.28	49.71 ± 1.11 ^ab^
SA 2.4 mM	3.96 ± 0.68	4.01 ± 0.62	41.03 ± 0.59	51.53 ± 2.01 ^ab^	46.12 ± 0.53	49.44 ± 5.52 ^ab^
BTH 0.6 mM	3.95 ± 0.44	4.19 ± 0.49	40.47 ± 1.52	46.38 ± 5.25 ^c^	47.13 ± 2.27	46.35 ± 1.49 ^b^
BTH 1.2 mM	3.80 ± 0.43	3.94 ± 0.71	40.93 ± 1.18	52.33 ± 3.02 ^a^	45.84 ± 0.89	52.26 ± 1.76 ^a^
BTH 2.4 mM	4.23 ± 0.74	4.04 ± 0.92	41.33 ± 0.33	53.09 ± 1.13 ^a^	47.34 ± 1.47	52.91 ± 3.38 ^a^
Inoc + non-treat	4.40 ± 0.41	4.27 ± 0.88	41.73 ± 0.71	44.62 ± 1.30 ^c^	46.73 ± 0.89	46.99 ± 2.64 ^b^
Non-Inoc	3.99 ± 0.61	4.09 ± 0.57	40.72 ± 0.48	42.95 ± 0.22 ^c^	46.25 ± 1.88	44.93 ± 1.56 ^b^
F-test	ns	ns	ns	**	ns	*
%CV	16.65	17.28	3.03	5.51	3.95	5.82

*Number of averages in before and after treated (n = 10).

**Figure 2:**
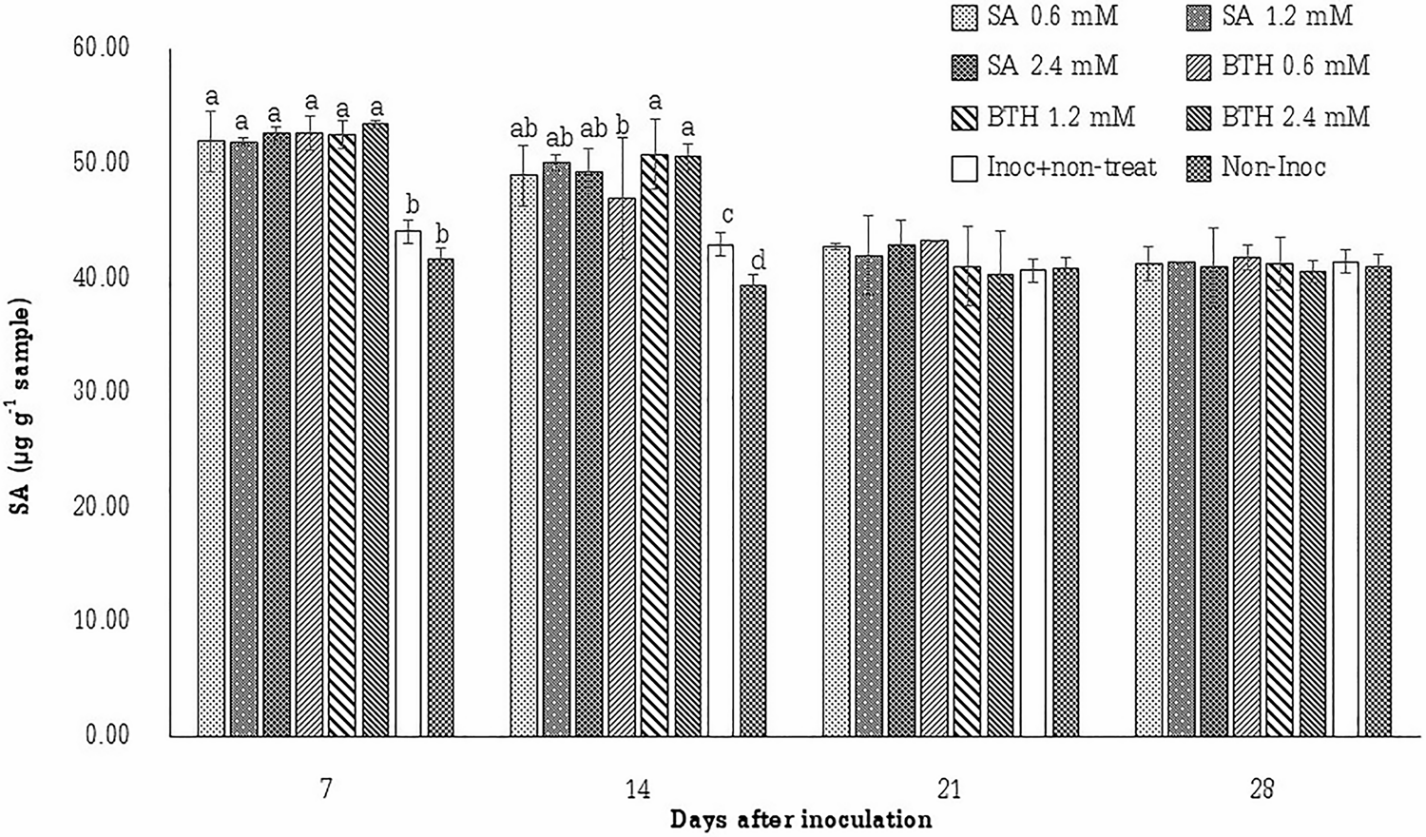
Quantification of salicylic acid (SA) in sugarcane leaves (µg g^−1^ sample) collected at 7, 14, 21, and 28 days after inoculation (n = 10 in each treatment).

The effect of elicitors on the synthesis of target biochemicals was studied. The SA content in the plants in both control and treatment groups before and after inoculation are given in Table 1, and Fig. 2, respectively. Before the application of treatments, the SA content in the control and treated plants did not show any statistical difference, while after spraying plants with SA and BTH for 4 weeks, the SA content of treated with SA and BTH significantly increased. In addition, plants treated with BTH at 1.2 mM and 2.4 mM showed the highest amounts of SA, followed by plants treated with SA at 2.4 mM, in comparison with the inoculated control. The Fig. 2 presents the SA content in sugarcane leaves after 7, 14, 21 and 28 days of inoculation. It was found that the treatments SA and BTH induced a rapid response in 7 days, while the treatment of BTH at 1.2 mM and 2.4 mM displayed the highest amounts of SA in 14 days, followed by the SA treatment at all concentration and BTH treatment at 0.6 mM, respectively. However, there was no significant difference detected in the SA content of all plants at 21 and 28 days after inoculation compared to the inoculated control. The treatments using BTH, and SA are thought to induce the production of SA in the plant.

**Figure 3:**
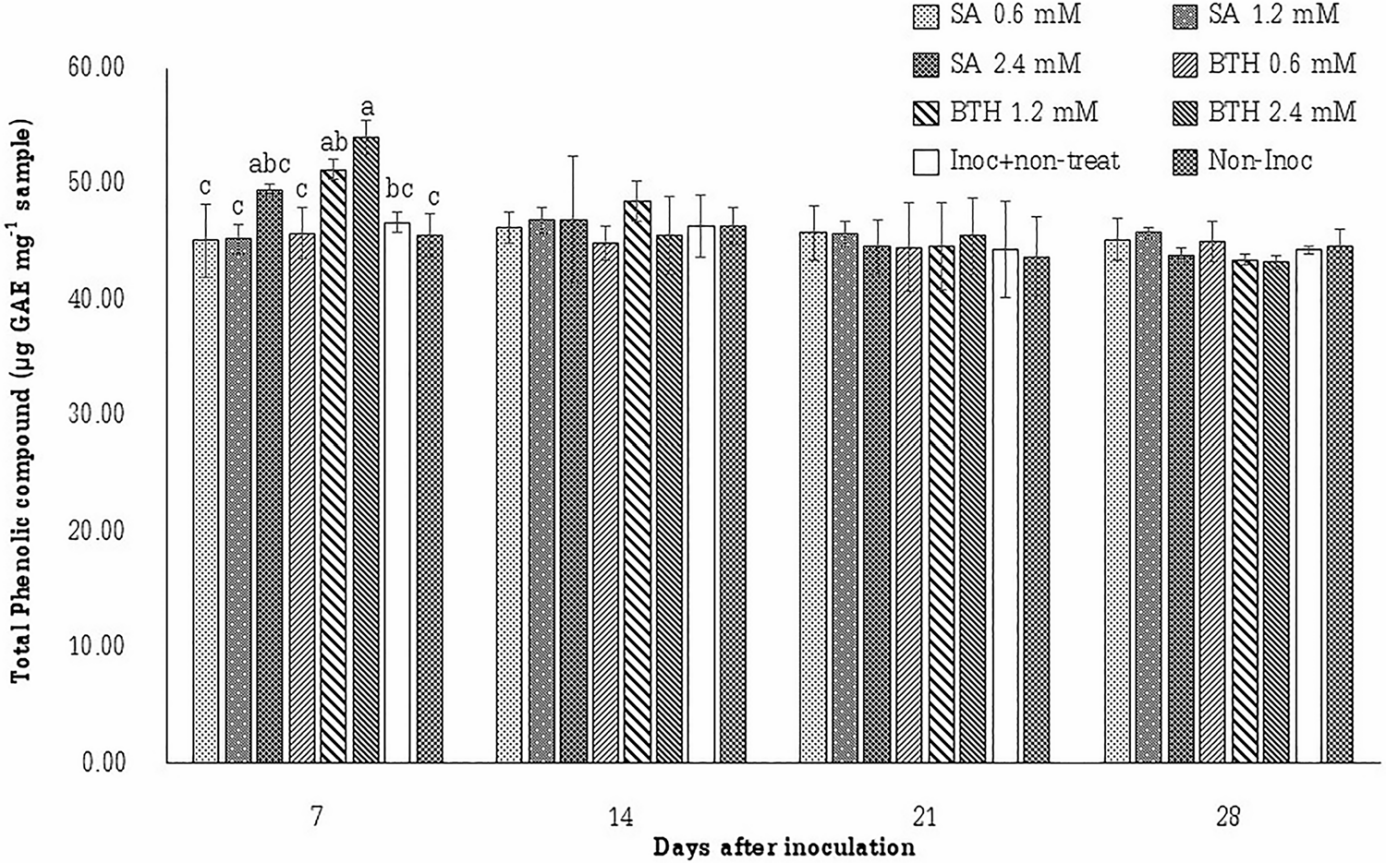
Quantification of total phenolic compound in sugarcane leaves (µg GAE mg^−1^ sample) collected at 7, 14, 21, and 28 days after inoculation (n = 10 in each treatment).

Additionally, the effect of elicitors on the content of total phenolic compounds in the plants is presented in Table 1. The content of total phenolic compounds in control and treated plants did not show any significant difference before spraying with BTH and SA. The same result was observed with the SA content. Furthermore, the treatment of BTH spraying at 1.2 and 2.4 mM showed the highest contents of total phenolic compounds. Meanwhile, the spray of SA at all concentrations revealed a tendency to increase the total Phenolic compound content in treated plants, however the increase was not significantly different in comparison with the inoculated control. At 7 days after inoculation (Fig. 3), the highest content of total phenolic compound in sugarcane leaves was achieved by the treatment BTH at 2.4 mM. There was a tendency to increase the content of total Phenolic compounds with the treatment using SA at 2.4 mM and BTH at 1.2 mM, however there was no significant difference noticed with the the inoculated control. The content of total phenolic compounds was not significantly different at 14, 21 and 28 days after inoculation.

Data was collected at timepoints 7, 14, 21, and 28 days after inoculation (n = 10 in each treatment).

## Discussion

This study demonstrated that spraying sugarcane plants with BTH and SA could stimulate the production of SA and phenolic compounds, hence helping treated plants to reduce the load of pathogen (concentration of phytoplasma). These results were similar with the previous reported on the application of BTH and Two glutathione-plus-oligosaccharide on grapes against the Bois noir^26^. In addition, the spraying of BTH solution at 4.8 mM reduced the infection from insect vectors in *Arabidopsis thaliana* by approximately 50%^24^, while the treatment using BTH at 2.4 mM was reported significant reductions of the disease severity in daisy plants. The application of SA at a concentration of 0.1 mM and 0.001 mM in potato that can against phytoplasma, decrease infection, high photosynthate translocation and improved high yield^34,35^. Moreover, the tomato can be against 53% of potato purple top phytoplasma infection after treated with SA^36^.

SA is a signaling molecule of the SAR in plants, SA could also induce the production of pathogenesis-related (PR) proteins involved in the defense mechanisms against several pathogens^22^. The SA also was reported induction of resistance in many crops such as sugarcane, rice, corn, and tomatoes^17,37^. Furthermore, the increase of PR proteins such as PR-1 in transgenic tobacco was correlated with lower infections by *P. parasitica* and *Peronosporatabacina*^38^.

Plants are often producing secondary metabolites such a phenolic compound to limit the development of the pathogen during an infection. The increase of total phenolic compounds in sugarcane leaves was found to be correlated with lower concentrations of phytoplasma in comparison with inoculated controls. This result similar the increase of tobacco plant resistance caused by the gene *VirA* and associated with the production of phenolic compounds^39^. Additionally, the high levels of phenolic compound in pearl millet could enhance the plant resistant of the fungus *Sclerospora graminicola*^40^.

The symptoms of white leaf disease were not observed during our experiment, even 1 month after the inoculation of plants. The expression of SCWL symptoms depends on several factors such as the age of the sugarcane, the number of phytoplasma cells, the temperature, and the subtropical climate^41^. In most cases, sugarcane present strong and severe SCWL symptoms in the ratoon stage.

Regarding our results, we conclude that spraying elicitors such as SA and BTH is able to induce a certain level of resistance associated with the SCWL by reducing the infectious load. The efficiency of elicitors was found to be related to the concentration of the substance. Nowadays, the control of the SCWL requires a variety of methods and the use of elicitors to stimulate the plant immune system is a promising strategy. We expect these results to be useful for the development of products aiming to control the SCWL in the future.

## Conclusion

Spraying sugarcane plants with BTH and SA could stimulate the production of SA and phenolic compounds, helping treated plants to reduce the load of pathogen (concentration of phytoplasma) after inoculation by insect vectors. This efficiency of elicitors was found to be related to the concentration of the substance. This research focused on increasing plant resistance against phytoplasma, reducing the infectious load and reducing the symptoms and severity of the SCWL. Nowadays, the control of the SCWL disease requires a variety of methods. The use of elicitors such as SA or BTH to stimulate the plant immune system is a promising strategy. We expect these results to be useful for the development of products aiming to control the SCWL disease in the future.

## Methods

### Plant material

Sugarcane (*Saccharum* spp. Hybrid, Khon Kaen 3) plants used for the experiment were from the collection of Mitr Phol Sugarcane Research Center. They were produced by tissue culture at Mitr Phol Sugarcane Research Center. Two-months old plantlets at the rooting stage were transplanted into 5-inch potting bags for 2 months and then transferred to 15-inch pots for 2 months.

### Insect vectors and phytoplasma transmission

To prepare the inoculation experiment, leafhoppers *(Matsumuratettix hiroglyphicus*) were collected in sugarcane fields located in the Khon Kaen Province of Thailand. In order to increase the population, captured insects were grown for 8 weeks in pots containing disease-free sugarcanes plants covered by a clear tube (15 cm in diameter and 30 cm in height). Insects were grown in controlled conditions at 25–29 °C and 70–80% relative humidity under natural light illumination according to a method adapted from previous studies^5,8^. Before the phytoplasma transmission, the F2 insect vectors were random checked by nested PCR to guarantee that its was disease free. To generate SCWL-infected vectors, F2 insect vectors were starved for 4 h and then fed by 12-weeks-old SCWL-infected sugarcane plant for 48 h. According to our nested PCR test experiment 80% of insect vectors were successfully inoculated by sugarcane phytoplasma after 48 h feeding on white leaf sugarcanes. This infection rate was commonly observed in previous experiments conducted on the transmission of phytoplasma to the insect vector *Matsumuratettix hiroglyphicus* at Mitr phol innovation and research center (unpublished works). The transmission of phytoplasma in sugarcane was performed. The 7 days after the fourth elicitors application of the treatment no. 2–8 (as shown in Table 2) were inoculated using F2 infectious leafhopper. Five adult insect vectors (2 male, 3 females) per pot were released in a clear plastic tube and maintained for 5 days in a controlled greenhouse. Then, insect vectors were removed without mortality during the transmission trials. This experiment used the vectors of *M. hiroglyphicus*, which were reported to more effective than *Y. flavovittatus *^5^. The temperature used for inoculation was between 25 and 29 °C, which was suitable for the transmission of phytoplasma. Five vectors were used per plant as a recognized and effective method for the inoculation of phytoplasma in sugarcane^42^.

**Table 2:** Description of elicitor treatments.

Treatment no	Control and treatments	Concentration (mM) of elicitor	Application
1	Non-Inoculated Control	–	30 mL of sterile distilled water/Time/Plot
2	Inoculated Control	–	30 mL of sterile distilled water/Time/Plot
3	Salicylic acid (SA)	0.6	30 mL/Time/Plot
4	Salicylic acid (SA)	1.2	30 mL/Time/Plot
5	Salicylic acid (SA)	2.4	30 mL/Time/Plot
6	Benzothiadiazole (BTH)	0.6	30 mL/Time/Plot
7	Benzothiadiazole (BTH)	1.2	30 mL/Time/Plot
8	Benzothiadiazole (BTH)	2.4	30 mL/Time/Plot

### Study on the efficiency of elicitor to stimulate resistance in control of SCWL and sampling

The experiment was conducted on 2-months old disease-free sugarcane plants following a Randomized Complete Block Design (RCBD) with ten replications, ten plants for each treatment. The control group was set in two types of a non-inoculated control and an inoculated control as shown in Table 1. The non-inoculated control referred to healthy plant while the inoculated control was infected plant. Both of them were not sprayed elicitors. Two types of elicitors treatment, SA and BTH were formulated in three different concentrations. BTH was dissolved in sterile distilled water while SA was first dissolved in a small amount of absolute ethanol and then diluted in sterile distilled water. Thirty milliliters of each elicitor treatment (Table 2) was applied to upper leaves of sugarcanes using a spray bottle, one time per day, every 7 day for four weeks. The controls of sugarcane pots were sprayed with an equivalent volume of sterile distilled water under the same conditions.

**Figure 4:**
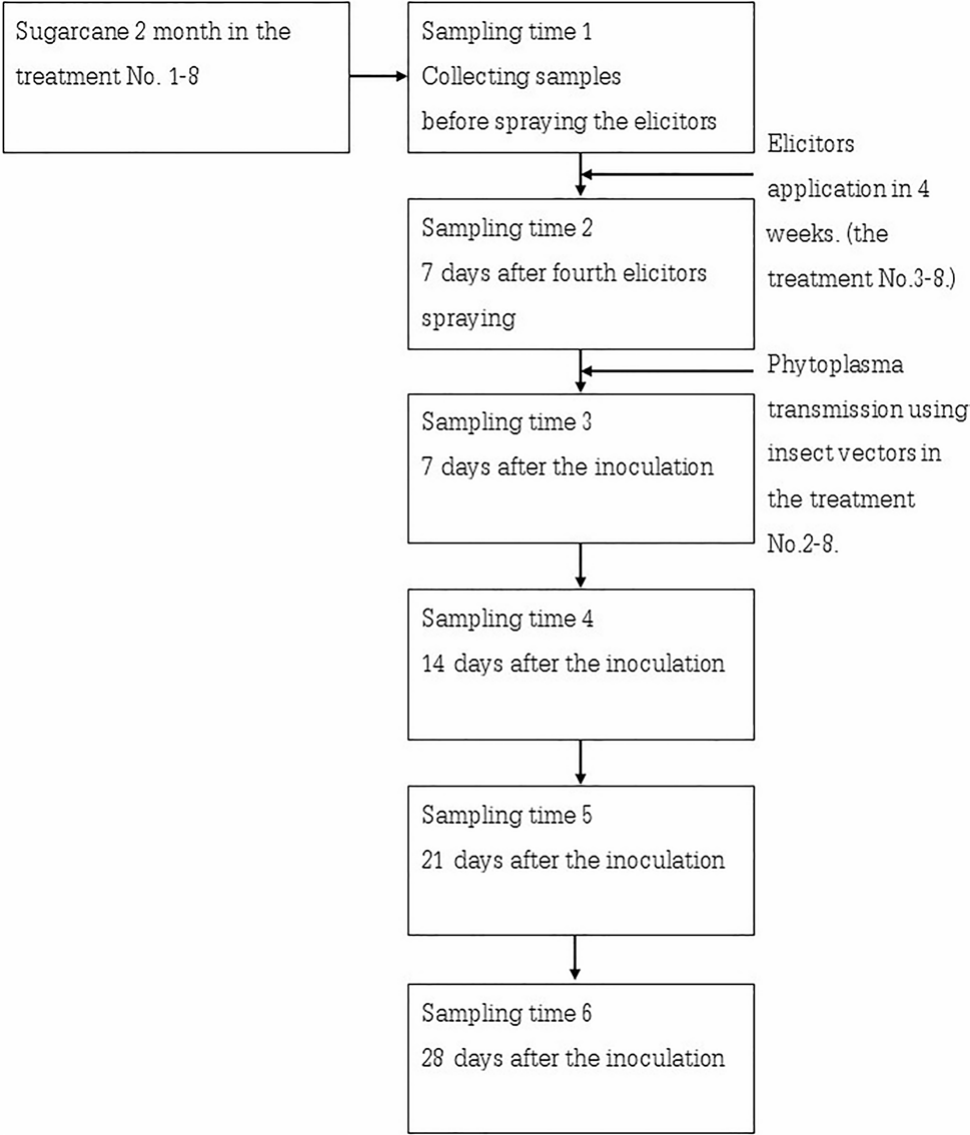
The collecting samples.

The collecting samples of this research was illustrated in Fig. 4. The healthy 2-months old sugarcane in each treatment was sampled to determine the amount of phytoplasma by RT-qPCR and analyzed for the quantification of SA and total phenolic compounds. The sampling time-1 was the original quantity of *secA* gene of phytoplasma, SA, and total phenolic compounds. The effect of elicitors on sugarcane to induce plants defense by comparing the analysis results in the sampling time 1 and 2. After inoculation in 7, 14, 21 and 28 days, sugarcane were sampled to determine.

### Quantification of phytoplasma

Total genomic DNA was extracted using a QIAamp 96 DNA Extraction Kit with a QIAcube HT automate (Qiagen, Germany). The diluted sample was quantified by NanoDrop ND-1000 Spectrophotometer (NanoDrop Technologies). All DNA samples were standardized by diluting to 100 ng/µL for use as template in the PCR reaction.

Extracted DNA solutions were used as templates for the amplification by PCR of the target phytoplasma gene *secA* and the reference sugarcane gene *18S SGC*^43,44^. Two primer sets were generated and used to amplify target DNA fragments as described in previous reported^43^ (Table 3). PCR reactions were performed using 1 µL of 100 ng/µL genomic DNA template in a 25 µL in total reaction volume containing 1xGreen Master Mix (Biotechrabbit, Germany), and 1 µM of each primer. The PCR condition was set up with a pre-denaturing step at 95 °C for 2 min followed by 35 cycles of denaturation 95 °C for 30 s, annealing 55.5 °C (*secA*), 59.5 °C (*18S SGC*) for 30 s and 72 °C for 45 s, with a final extension of 72 °C for 5 min.

**Table 3:** List of primers and their sequences used in PCR reaction.

Target gene	Primer	Sequence	Product
*secA*	SecAfor	5’-GTTTTATATGGATGCTAATCGTTTT-3’	275 bp
	SecArev	5’-CTAYTGTTCTTCCTGTAAATTGATC-3’	
*18 s SGC*	18 s SGCf	5’-CCTTAGGCGTCAAGGAACAC-3’	201 bp
	18 s SGCr	5’-GCGTTCAAAAACTCGATGGT-3’	

PCR products were purified with a GenepHlow Gel/PCR Kit (Geneaid Biotech, Taiwan) following the method specified. The DNA fragments were then ligated with RBC TA cloning vector (RBCBioscience, Taiwan) to yield RBC-SecA and RBC-18S SGC plasmids. The plasmids were introduced into *Escherichia coli* Top10 competent cells (Thermo Fisher Scientific, USA) according to the method specified in the kit and incubated in LB solid media (Himedia, India) containing 50 mg/mL ampicillin at 37 °C, overnight. The plasmid DNA was extracted from the selected colonies using blue-white selection and assayed for the presence of the target. The plasmids were purified from overnight cultures of the transformed *E. coli* cells using GenUP Plasmid Kit (Biotechrabbit, Germany). Plasmid concentrations were estimated using a NanoDrop ND-1000 Spectrophotometer (NanoDrop Technologies) and stored at − 20 °C. The number of target DNA copies in the plasmid solution was calculated based on the plasmid and insert size, according to the following formula:

$${\text{Number}}\;{\text{of}}\;{\text{copies }} = \frac{{\left[ {{\text{amount}}\; {\text{of}}\;{\text{ DNA}}\; \left( {\frac{{{\text{ng}}}}{{{\mu l}}}} \right) \times \left( {6.022 \times 1023} \right)} \right]}}{{\left[ {{\text{Length}}\; \left( {{\text{bp}}} \right) \times \left( {1 \times 109 \times 650} \right)} \right]}}$$

The plasmids were then serially diluted in 1 × TE buffer (pH 8.0), and plasmid DNA concentrations of 10^8^–10^2^ copies were used to plot the standard curve for qPCR, with the PCR amplification efficiency (E) equal to 2 (doubling the amount of DNA in each cycle).

The concentration of phytoplasma present in our DNA samples was measured by RT-qPCR using the plasmids of each target gene as a reference for the standard curve. Each sample was replicated. qPCR reactions contained 100 ng of DNA template, 1X Quantinova SYBR Green PCR Master Mix (Qiagen, Germany), 0.7 µM Forward Primer, 0.7 µM Reverse Primer, for a total volume of 20 µL. The reactions were performed with a Rotor-Gene Q Real-Time PCR System (Qiagen, Germany). Reaction parameters consisted of initial-denaturing at 95 °C for 2 min, followed by 40 cycles of denaturing at 95 °C for 5 s and annealing at 60 °C for 10 s. Results were calculated and analyzed with the Q-Rex Software (Qiagen, Germany).

### Extraction and quantification of salicylic acid

Free SA extraction was performed as described previously^45^. The sugarcane leaf samples were cleaned and stored at 4 °C. Leaf samples were cut into small pieces of 1 cm x 1 cm size, 0.5 g were ground in liquid nitrogen then dissolved in 0.5 mL of 90% (v/v) methanol. The sample solution was transferred to a 1.5 mL micro-tube and centrifuged at 12,000 rpm for 20 min.

For the quantification of SA, 150 µL of supernatant was transferred to 96 well plates with 150 µL of 0.02 M ferric ammonium sulfate and incubated at 30 °C for 5 min. Free SA was detected using a spectrophotometer at the emission wavelength 530 nm. The standard curve was prepared by serial dilutions of a salicylic acid standard solution.

### Extraction and quantification of phenolic compounds

For the quantification of phenolic compounds, 20 µL of supernatant of sugarcane leaf extract was transferred to 96 well plates with 100 µL of 10% Folin-Ciocalteu reagent and 80 µL of 7% sodium carbonate (Na_2_CO_3_) and then incubated at room temperature for 30 min. Total phenolic compounds were evaluated using a spectrophotometer at the emission wavelength of 760 nm. The standard curve was prepared by serial dilutions of a 1 M gallic acid standard solution. (Results were reported as µg GAE (Galic acid equivalent) per mg. of sample^46^.

### Statistics

A RCBD with ten replications were used in this experiment. The average and standard error of quantification of *secA* gene of phytoplasma, SA values and total phenolic compounds values from ten replications in each treatment were calculated using Microsoft Excel. One-way analysis of variance (ANOVA) and LSD test were used for considering the differences among treatments statistically significant at p-value < 0.05 using STAT8^47^.

### Compliance with Ethical Standards

All the experiment has been done in the accordance with the relevant institutional, national, and international guidelines /legislation.

## Supplementary Information

Supplementary Information.
