# Supplementary material for: The role of salicylic acid and benzothiadiazole in decreasing phytoplasma titer of sugarcane white leaf disease
Source: Sci Rep. 2021 Jul 26;11:15211. doi: 10.1038/s41598-021-94746-9 (PMC8313703; doi:10.1038/s41598-021-94746-9)
Supplement: Supplementary file 1 — Supplementary Information. [file 41598_2021_94746_MOESM1_ESM.pptx]

## Slide 1
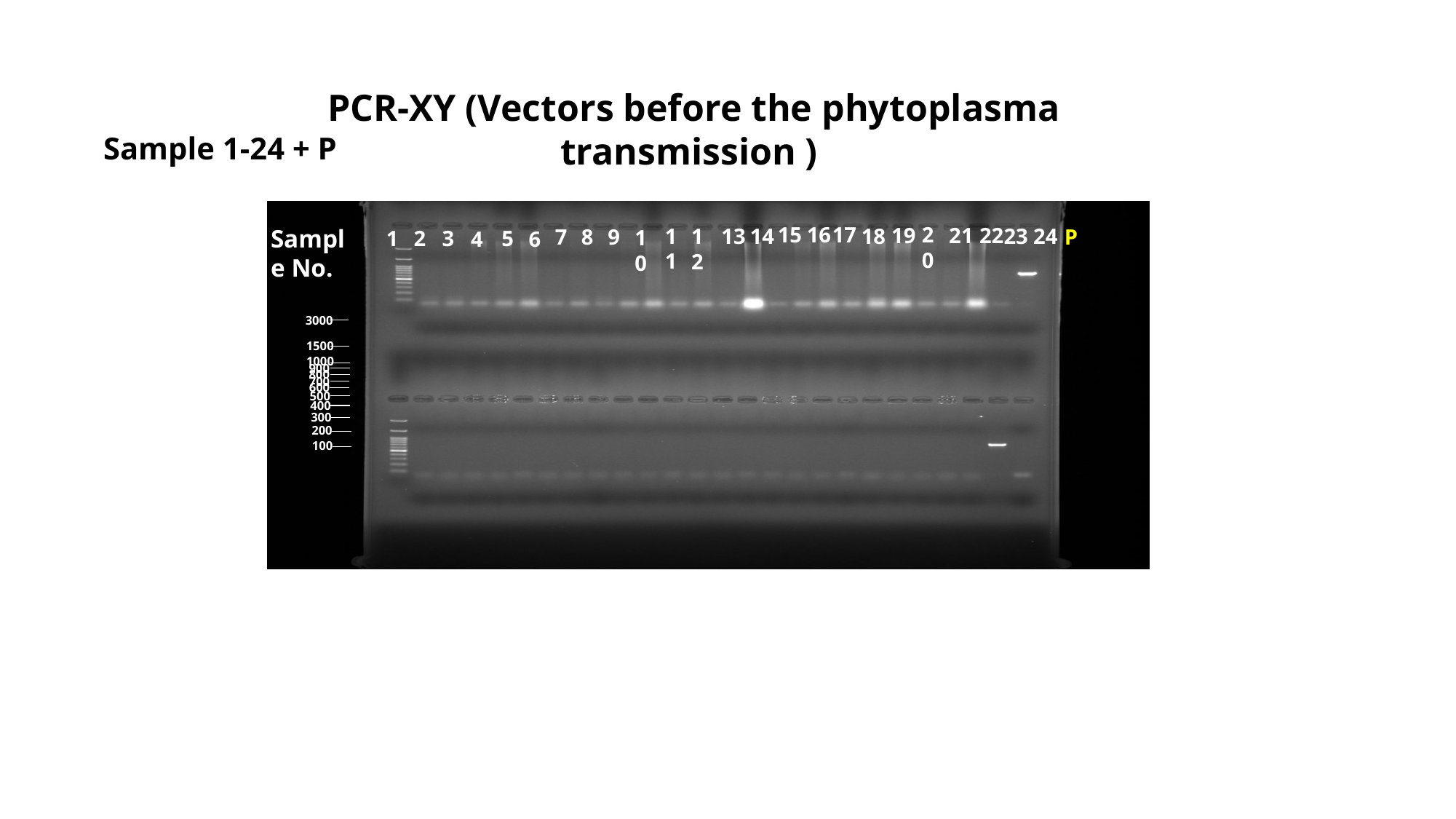

PCR-XY (Vectors before the phytoplasma transmission )
Sample 1-24 + P
20
15
16
17
19
21
22
11
18
23
12
14
13
24
7
8
9
Sample No.
P
10
5
1
2
3
4
6
3000
1500
1000
900
800
700
600
500
400
300
200
100

## Slide 2
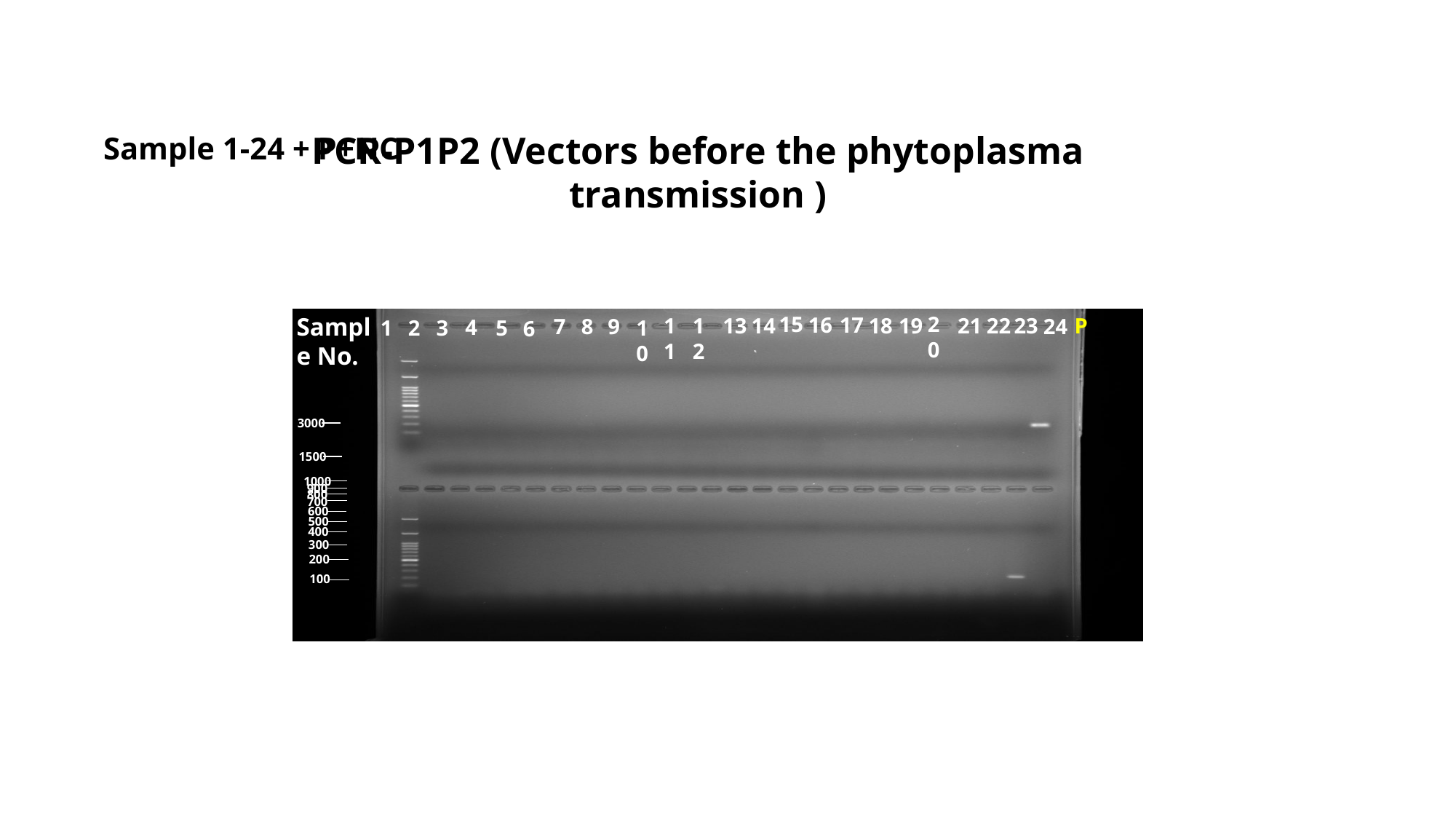

PCR-P1P2 (Vectors before the phytoplasma transmission )
Sample 1-24 + P+NC
20
15
16
17
Sample No.
19
21
22
11
P
18
23
12
14
13
24
7
8
9
4
10
5
1
2
3
6
3000
1500
1000
900
800
700
600
500
400
300
200
100

## Slide 3
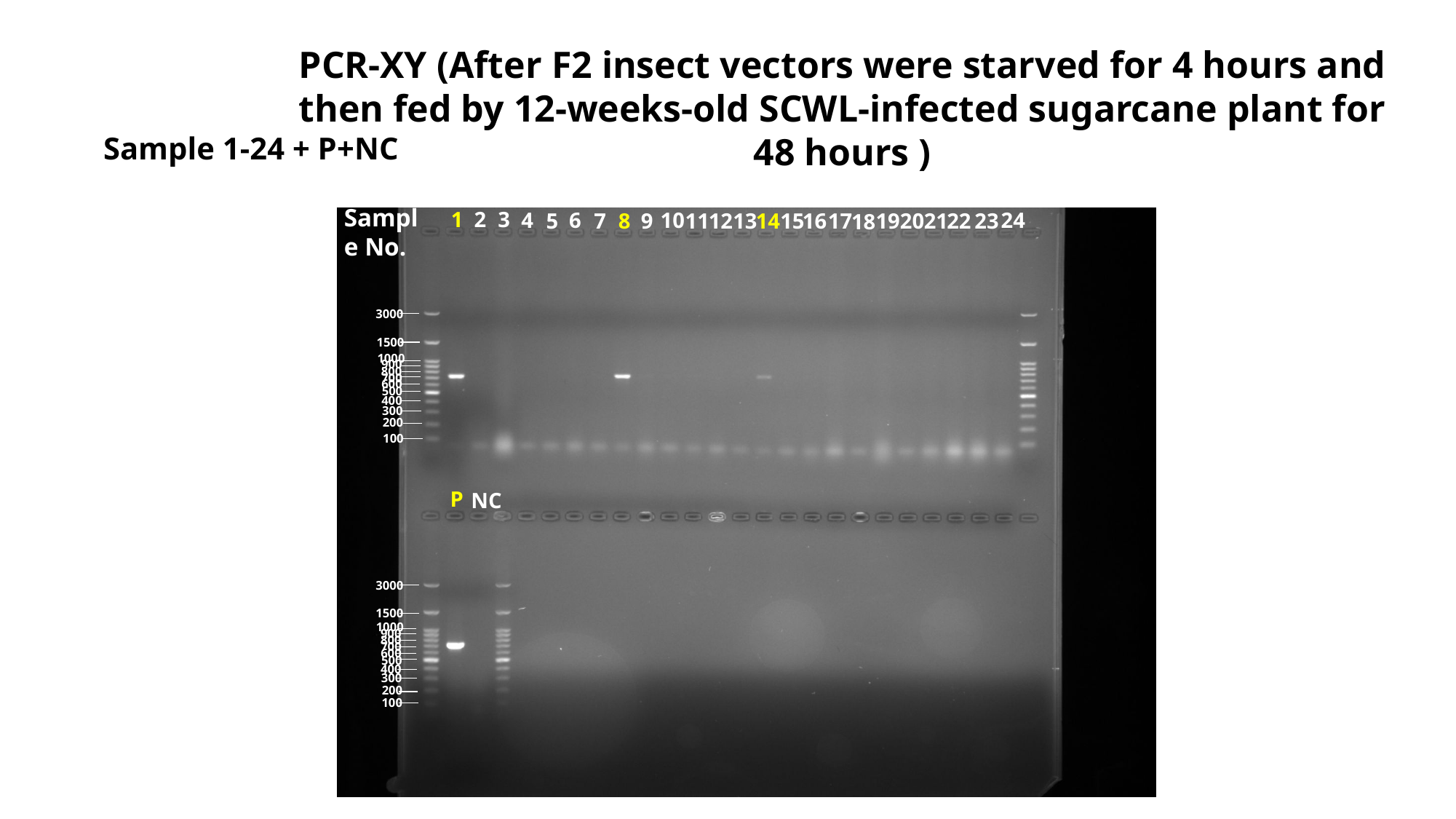

PCR-XY (After F2 insect vectors were starved for 4 hours and then fed by 12-weeks-old SCWL-infected sugarcane plant for 48 hours )
Sample 1-24 + P+NC
Sample No.
1
2
3
4
24
6
10
5
16
17
23
20
11
13
14
15
12
21
9
7
8
22
19
18
3000
1500
1000
900
800
700
600
500
400
300
200
100
P
NC
3000
1500
1000
900
800
700
600
500
400
300
200
100

## Slide 4
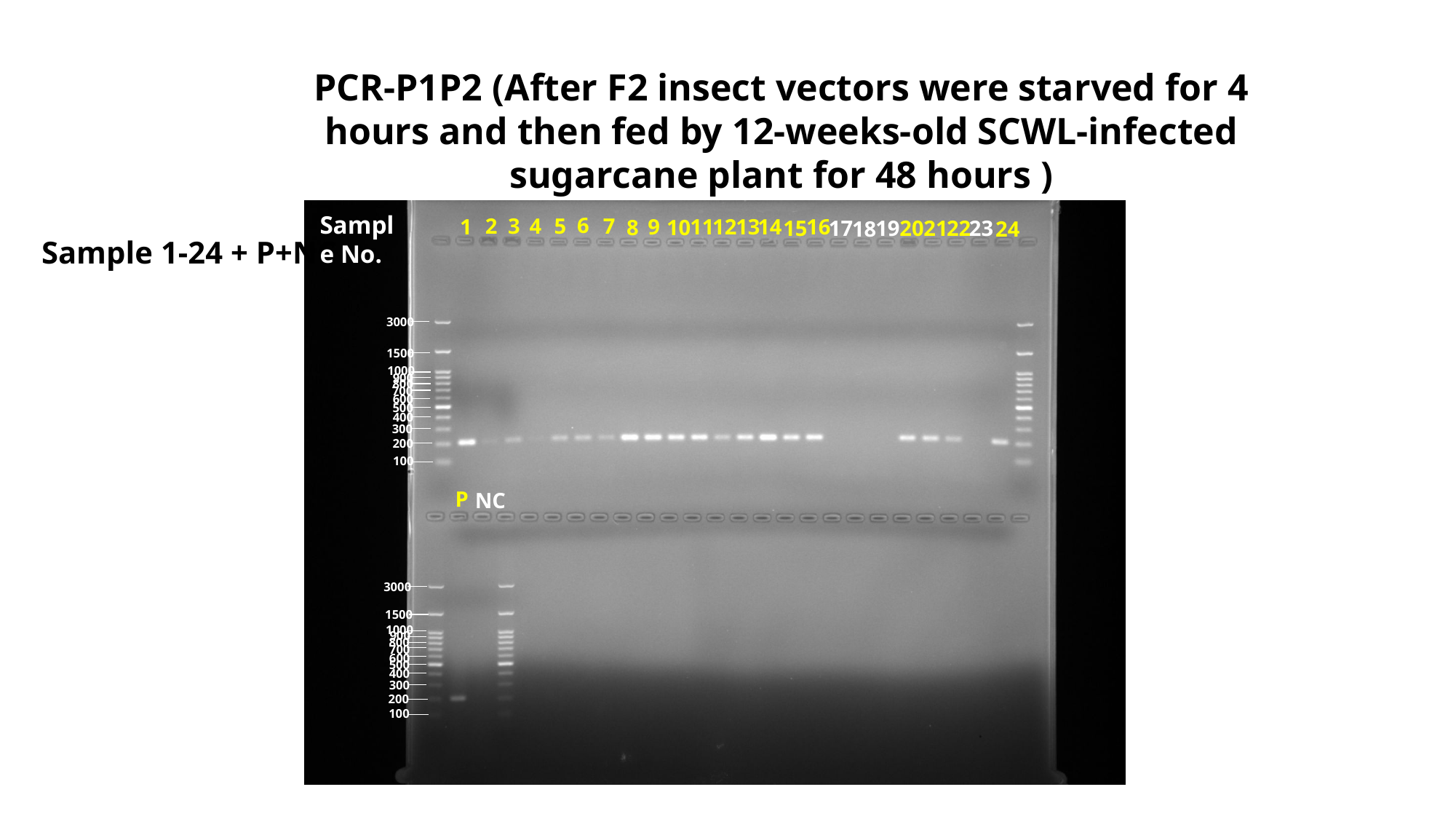

PCR-P1P2 (After F2 insect vectors were starved for 4 hours and then fed by 12-weeks-old SCWL-infected sugarcane plant for 48 hours )
Sample No.
6
5
2
3
7
4
1
16
11
12
13
14
9
10
8
17
20
15
21
22
23
19
24
18
Sample 1-24 + P+NC
3000
1500
1000
900
800
700
600
500
400
300
200
100
P
NC
3000
1500
1000
900
800
700
600
500
400
300
200
100
